# ADAM8 promotes alcoholic liver fibrosis through the MAPK signaling pathway

**DOI:** 10.1186/s12576-024-00943-2

**Published:** 2024-10-16

**Authors:** Mengli Yang, Sanqiang Li, Renli Luo, Yadi Zhao, Yue Sun, Haoyuan Li, Qinyi Cui, Junfei Wu, Longfei Mao

**Affiliations:** 1 The Molecular Medicine Key Laboratory of Liver Injury and Repair, College of Basic Medicine and Forensic Medicine, Henan University of Science and Technology, 263 KaiYuan Road, Luoyang, 471000 Henan China; 2 Henan Center for Engineering and Technology Research on Prevention and Treatment of Liver Diseases, Luoyang, 471000 Henan China

**Keywords:** ADAM8, Alcoholic liver fibrosis, MAPK signaling pathway, Hepatic stellate cells

## Abstract

The effect and molecular regulatory mechanism of A Disintegrin and Metalloproteinase 8 (ADAM8) were explored in alcoholic liver fibrosis (ALF). C57BL/6N male mice were randomly divided into control, alcohol, and ADAM8-sgRNA3 plasmid groups. The control group received control liquid diet, while the alcohol and ADAM8-sgRNA3 plasmid groups were given alcohol liquid feed diet combined with ethanol gavage treatment for 8 weeks to induce ALF modeling. In addition, the ADAM8-sgRNA3 plasmid group was injected with the effective ADAM8-sgRNA3 plasmid, while the alcohol and control group mice were injected with an equivalent amount of physiological saline. LX-2 human hepatic stellate cells were divided into control, alcohol, si-ADAM8-2, and si-ADAM8-NC groups and induced for 48 h for model establishment in vitro. Serological detection, pathological staining, Western blotting, qRT-PCR and CCK8 assay were performed for experiments. Compared with the alcohol group, ADAM8 mRNA, protein and, positive area rate, serological indicators, pathological changes, and the expression of liver fibrosis marker and MAPK signaling pathway-related factors in the ADAM8-sgRNA3 plasmid group significantly decreased in vivo. Compared with the alcohol group, ADAM8 mRNA and protein expression, cell viability, and the expression of liver fibrosis markers and MAPK signaling pathway-related factors (p-ERK1/2, PCNA, Bcl-2, p-c-Jun, TGFβ1, p–p38 MAPK and HSP27) reduced significantly in the si-ADAM8-2 group. Therefore, ADAM8 promotes ALF through the MAPK signaling pathway, a promising target for treating ALF.

**Supplementary Information:**

The online version contains supplementary material available at 10.1186/s12576-024-00943-2.

## Background

In recent years, drinking has become a common habit worldwide, which has had a major impact on physical and mental health. Alcohol is classified as a Class I carcinogen and associated with over 200 diseases. The liver is the organ that suffers the most extensive tissue damage, and over 75 million people worldwide are at risk of alcohol-related liver disease [1, 2]. Approximately 2 million people die from alcoholic liver disease each year. Long-term excessive drinking results in liver disease progression from fatty liver and hepatitis to alcoholic liver fibrosis (ALF), and can even lead to cirrhosis and liver cancer [3]. ALF refers to the pathological process of the repair of liver inflammation or liver tissue damage during continuous alcohol stimulation [4]. Studies have shown that the timely elimination of injury factors as well as active and effective treatment can slow down or even reverse the process of liver fibrosis [5, 6], which is important for improving patient quality of life. However, there is currently no specific treatment for ALF besides alcohol withdrawal, nutritional support, and anti-fibrotic drug therapy [7]. Therefore, there is urgent to identify effective targets and related mechanisms to treat this disease.

Under the long-term action of ethanol and its primary metabolite acetaldehyde, the extracellular matrix (ECM) components of the liver are excessively deposited [8]. Hepatic stellate cells (HSCs) are the main source of ECM and a key target of liver fibrosis [9, 10]. The activation of HSC has an important role in the initiation, development, and reversal of liver fibrosis [11, 12]. Alcohol can stimulate changes in the phenotype of HSCs and activated HSCs are converted into myofibroblasts, secreting a large amount of α-smooth muscle actin (α-SMA) and collagen fibrin, which in turn, generates a large amount of ECM and deposit in the liver [13, 14], which leads to liver fibrosis [15]. Therefore, preventing the activation of HSC is an effective strategy for treating liver fibrosis.

A disintegrin and metalloprotease 8 (ADAM8) with 8 structural domains is an important member of the ADAM family that exhibits metal endoprotease activity and disintegrin function. ADAM8 is widely involved in the hydrolysis of a series of membrane-binding receptors, cytokines, and other proteins, as well as in regulating cell morphology and movement [16]. It has multiple ligands that are involved in various signaling pathways to jointly regulate intracellular signal transduction and physiological function [17]. Under pathological conditions, high expression of ADAM8 is closely associated with the inflammatory response of various diseases [18], damage to tissues and cells, ECM remodeling [19], and various signaling pathways. This suggests that ADAM8 may be a potential biomarker in the development of liver fibrosis. Preliminary studies in our laboratory indicate that in acute liver injury induced by 50% (v/v) ethanol (14 mL/kg) in mice, the ADAM8 expression is significantly increased, thereby promoting liver damage. However, the specific role and molecular mechanism of ADAM8 in the progression of ALF is unknown.

In this study, an alcoholic liquid diet combined with 31.5% ethanol gavage was administered to mice to establish an in vivo model of alcoholic liver fibrosis. CRISPR/Cas9 technology was used to target and inhibit the expression of ADAM8 to examine the effect of downregulating ADAM8 on ALF in mice. In vitro experiments, a 50 mM ethanol culture medium was used to stimulate the activation of human hepatic stellate cell LX-2. The expression of ADAM8 was downregulated by siRNA to identify the underlying mechanism of ADAM8 in ALF.

## Methods

### Design and synthesis of an effective plasmid to inhibit the expression of the ADAM8 gene in mice by CRISPR/Cas9 technology

Based on the ADAM8 gene information in the Ensemble database of the NCBI website (https://www.ncbi.nlm.nih.gov/gene/11501), there is no continuous homologous CDS sequence between mice and humans. Therefore, we designed a CRISPR sequence to inhibit the expression of the ADAM8 gene in mice. Based on the analysis of the conserved structural and functional domains of the protein, Exon 5 was selected as the site to be knocked out. Three single-guide RNAs (sgRNAs) were designed and synthesized to increase the probability of multisite cleavage and improve knockout efficiency, namely ADAM8-sgRNA1: CCTAGCTGCATCCCGCTCCCGCA, ADAM8-sgRNA2: AGTATGAGTGGTTGGCCCGG, and ADAM8-sgRNA3: CCCCTGGACCTCTTGCCCCAT.

In this study, incomplete knockout of the ADAM8 gene in C2C12 cells by RNP gene knockout technology was intended to facilitate subsequent sequencing to identify the most effective sgRNA, which was obtained from the Boster Biological Technology Co., Ltd (Wuhan, China). Digest the cells, wash the cells twice with DPBS solution and prepare the appropriate number of cells for the experiment. The complex formed from Cas 9 and sgRNA at a ratio of 5:1 to 10:1 for 10 min was electrotransferred into C2C12 cells, according to the instructions provided with the Thermo Fisher Neon Transfection System. The electroporation conditions are shown in Table 1.

**Table 1:** Electric conversion conditions

Pulse voltage(v)	Pulse width (ms)	Pulse number	Cell density (cells/ml)	Transfection efficiency	Viability	Tip type
1650	10	3	5 × 10⁶	95%	96%	10 μL

Then, procee with the screening of effective ADAM8-sgRNA. Culture the cells for 3–5 day post-electroporation until the cell density in the 24-well plate reaches more than 60%. Discard the medium, wash the cells with PBS once, then add 50 μL of lysis buffer to each well. Pipette the cells to form a suspension. Simultaneously, wild-type C2C12 cells were also treated to form a suspension as a control. The two cell suspensions were sequenced and compared to screen for the effective ADAM8-sgRNA. Further validation of the screened effective ADAM8-sgRNA was done by protein immunoblotting. The observed phenotypic changes allowed us to explore the biological functions of reduced ADAM8 in a more nuanced manner.

Corresponding complementary primers were designed and synthesized based on the selected effective ADAM8-sgRNA sequences (F: CACCGGATGGGGCAAGGGGGGTCTCTTGCCCCATCC, R: AAACCTGGACCTTTTTTCCCATCC). The effective three-in-one recombinant plasmid (simultaneously expressing sgRNA, Cas9, and puro) was constructed to inhibit the mouse ADAM8 gene by CRISPR/Cas9 technology, designated pYSY-CMV-Cas9-U6-ADAM8-sgRNA-EFla-puro, and purchased from Nanjing Yaoshunyu Biotechnology Co., Ltd (Nanjing, China). PCR and sequence validation of the transformants were conducted.

### Animals and ethics considerations

Specific pathogen-free male C57BL/6N mice (SCXK20190001), weighing 22 ± 2 g and aged 6–8 weeks, were acquired from the Zhejiang Vital River Laboratory Animal Technology Co., Ltd (Beijing, China). The mice were raised in the animal facility at the College of Basic Medicine and Forensic Medicine, Henan University of Science and Technology, with good ventilation, humidity (50% ± 10%), temperature (25 ± 2 ℃), and a 12 h light/dark cycle. The mice were provided with standard water and feed ad libitum. After 1 week of acclimatization, modeling began. All procedures were performed in compliance with the Guide for the Care and Use of Laboratory Animals. The protocols for animal experiments received approval from the Institutional Ethical Committee of Henan University of Science and Technology.

### Mouse model of ALF

30 mice were randomly assigned to three groups, each consisting of 10 mice: a control group, an alcohol group, and an ADAM8-sgRNA3 plasmid group. The mice in the control group were given a TP4060-28AC control liquid diet (protein 22.5%, fat 44%, carbohydrates 33.5%) obtained from Trophic Animal Feed High-Tech Co., Ltd (Nantong, China), whereas the alcohol and ADAM8-sgRNA3 plasmid groups were given a TP4060-28A alcohol liquid feed diet (protein 22.5%, fat 44%, carbohydrates 5.5%, alcohol 28%) combined with 31.5% ethanol gavage treatment (5 g/kg, twice a week) for 8 weeks to induce ALF modeling [20]. The mice in the ADAM8-sgRNA3 plasmid group were injected with the effective ADAM8-sgRNA3 plasmid (2 g/kg, twice a week) through tail vein injection, whereas the alcohol and control group mice were injected with an equivalent amount of physiological saline. On the 8th weekend, all mice were fasted for 24 h and euthanized with CO_2_ exposure.

### Detection of serum ALT and AST activity and TNF-α and IL-1 concentration

Blood samples were collected from the eyeballs of the mice and allowed to coagulate naturally at room temperature for 20 min. Serum was obtained by centrifugation (4000 rpm, 20 min) and promptly used to measure the activity of ALT and AST using assay kits procured from Nanjing Jiancheng Bioengineering Institute (Nanjing, China). TNF-α and IL-1 were measured using assay kits procured from Quanzhou Ruixin Biotechnology Co., Ltd (Quanzhou, China).

### Histopathological analysis

The mice were sacrificed, liver tissues were harvested, and paraffin and frozen sections were prepared from each group. The changes of fibrosis severity, ADAM8 protein expression, injury situation, glycogen content, and lipid droplet content in the liver tissue were examined by Sirius Red staining, immunohistology staining, hematoxylin–eosin (H&E) staining, Periodic Acid-Schiff (PAS) staining, and Oil Red O staining, respectively. The Sirius Red and PAS staining kits were procured from Beijing Jinclone Biotechnology Co., Ltd (Beijing, China), whereas H&E and Oil Red O staining kits were obtained from Biosharp Life Sciences Co., Ltd (Hefei, China) and Wuhan Servicebio Technology Co., Ltd (Wuhan, China), respectively. The immunohistology staining kit was purchased from Beijing Zhongshan Jinqiao Biotechnology Co., Ltd (Beijing, China).

### Detection of ADAM8 mRNA in liver tissue by qRT-PCR

Total RNA was extracted from the liver tissue of each mouse using the total RNA rapid extraction kit from Shanghai Feijie Biotechnology Co., Ltd (Shanghai, China). RNA concentration was measured using a Micro UV–Vis spectrophotometer (Thermo Fisher, Munich, Germany). The RNA was reverse-transcribed into cDNA using the ReverTra Ace qPCR RT Kit from TOYOBO Biotech CO., Ltd (Shanghai, China). Bestar® SybrGreen qPCR master mix from Shanghai Xinghan Sci&Tech Co., Ltd. (Shanghai, China) was used for qRT-PCR with the following primer sets: ADAM8-forward: GTAGGTTAAACAGGCACT, ADAM8-reverse: CACAGGAGGACCATCGCCAT; β-actin-forward: TTCCTTGGTAGGAAT, β-actin-reverse: GAGCAATGATCGATCGATCGATCC. The primer sequences were designed and synthesized by the Beijing Genomics Institute (Beijing, China). After amplification using a quantitative PCR instrument (Applied Biosystems, USA), the relative expression of ADAM8 mRNA was determined using the 2^−△△Ct^ method and normalized to β-actin gene expression.

### Cell culture

The human hepatic stellate cell LX-2 commonly used in liver fibrosis research was procured from Shanghai Zhongqiao Xinzhou Biotechnology Co., Ltd (Shanghai, China) and cultured in Dulbecco’s Modified Eagle Media (DMEM) high glucose medium containing 10% (*v*/*v*) fetal bovine serum (FBS) and 1% (*v*/*v*) penicillin–streptomycin. The DMEM medium and FBS were obtained from Gibco (Gaithersburg, MA, USA) and Biological Industries (Zhejiang, China), respectively. Cell culture was carried out under general culture conditions of saturated humidity, 37 ºC, and 5% CO_2_.

### Determining the optimal ethanol concentration for activating LX-2 cells by cell counting kit 8 (CCK8) assay

LX-2 cells (100 µl) were seeded at approximately 2 × 10^3^ cells per well in a 96-well plate. After the cells fully adhered, the original culture medium was replaced with a fresh culture medium containing different concentrations of ethanol (0, 25, 50, 100, 200, and 400 mM). After 48 h, the medium was replaced with 100 µl of culture medium containing 10 µl of CCK-8 solution from Biosharp Life Sciences Co., Ltd (Hefei, China). After a 1 h incubation, the absorbance values at 450 nm were measured using an ELISA plate reader (Bio-Tek, USA). Cell viability was determined as follows: Cell viability = [OD (alcohol) − OD (blank)]/[OD (0 alcohol) − OD (blank)] × 100%.

### Screening of si-ADAM8

LX-2 cells were seeded into a 6-well plate and randomly divided into control group, alcohol group, si-ADAM8-1 group, si-ADAM8-2 group, si-ADAM8-3 group, and si-ADAM8-NC group. Once the cells reached 30–50% confluence, the control group was cultured normally and the remaining five groups were treated for 48 h with 50 mM ethanol to establish an ALF model in vitro. Except for the control group and the alcohol group, the other four groups were transfected with si-ADAM8-1, si-ADAM8-2, si-ADAM8-3, and si-ADAM8-NC, respectively. The siRNAs were purchased from Shanghai GenePharma Co., Ltd (Shanghai, China), and the sequences are provided in Table 2. Transfection was done using the RiboFET CP transfection reagent from Guangzhou RiboBio Co., Ltd (Guangzhou, China). RNA was extracted from LX-2 cells and subject to qRT-PCR to measure the expression of ADAM8 mRNA and to determine the optimal si-ADAM8. The following primer sets were used: ADAM8-forward: GCTGATGTGGTGTGGACA, ADAM8-reverse: CAGGACCACCGGAGAGTTGGA; GAPDH-forward: CATGAAGATTATGACACGCCT, GAPDH-reverse: AGTCTTCCACGATACAACAAGT.

**Table 2:** Sequence of si-ADAM8-1, si-ADAM8-2, si-ADAM8-3, and si-ADAM8-NC

	Sense(5–3ʹ)	Antisense(5–3ʹ)
si-ADAM8-1	GCCAGGACUUACACGUUUATT	UAAACGUGUAAGUCCUGGCTT
si-ADAM8-2	GGACAAGCUAUAUCAGAAATT	UUUCUGAUAUAGCUUGUCCTT
si-ADAM8-3	GCAUGGACCAUGAUGAGAATT	UUCUCAUCAUGGUCCAUGCTT
si-ADAM8-NC	GCGACGAUCUGCCUAAGAUDTDT	AUCUUAGGCAGAUCGUCGCDTDT

### In vitro model of ALF in LX-2 cells

The cells were categorized into the following four groups: a control group, an alcohol group, a si-ADAM8-2 group, and a si-ADAM8-NC group. Except for the cells in the control group cultured under standard conditions, LX-2 cells in the remaining four groups were treated with 50 mM ethanol for 48 h.

### Detection of proliferation activity of LX-2 cells in each group by CCK8

LX-2 cells were seeded into a 96-well plate. After fully adhering, the cells were treated according to their corresponding group. After 48 h of transfection and co-treatment with ethanol, the CCK8 assay was used to measure the proliferation of LX-2 cells in each group based on the following formula: Cell viability = [OD (intervention) − OD (blank)]/[OD (control) − OD (blank)] × 100%

### Western blot analysis

Total protein concentration extracted from the LX-2 cell and liver tissue was determined and a Western blot analysis was conducted. Primary antibodies against PDGF-B, ADAM8, p-ERK1/2, PCNA, TNF-α, p-c-Jun, p–p38 MAPK, and HSP27 were purchased from Santa Cruz Biotechnology, Inc. (USA). α-SMA antibody was from Wailei Biotechnology Co., Ltd (Chenyang, China), Collagen-I antibody was from Proteintech Group, Inc (Wuhan, China), TGF-β1 antibody was from Zhengneng Biotechnology Co., Ltd (Chengdu, China), GAPDH and Bcl-2 antibodies were from Hua'an Biotechnology Co., Ltd, and HRP-conjugated goat anti-mouse or rabbit antibodies were purchased from Beijing Abway Antibody Technology Co., Ltd (Beijing, China).

### Statistical analysis

The data are presented as the mean ± standard deviation and were subjected to statistical analysis using Prism GraphPad 9.5.0. Comparison between multiple groups was done using Tukey's post hoc test.

## Results

### Successful construction of the effective plasmid to inhibit mouse ADAM8 gene

**Fig. 1:**
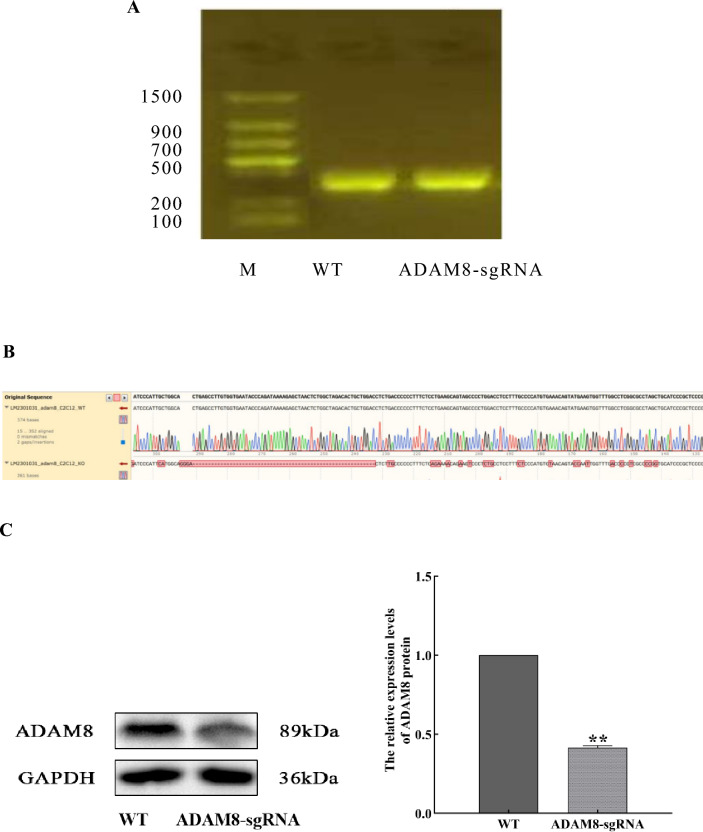
Successful construction of ADAM8-sgRNA3 plasmid for inhibiting murine ADAM8 gene expression. **A** Agarose gel electrophoresis. WT: wild-type C2C12 cells, ADAM8-sgRNA: C2C12 cells transfected with the ADAM8-sgRNA, and M: molecular weight marker. **B** Genotype comparison results between wild-type and mutant cells. **C** Immunoblotting and quantitative analysis of ADAM8 protein expression in both wild-type and ADAM8-sgRNA cells (compared with WT, ^**^P < 0.01)

The sgRNA and Cas9 complex were electroporated into C2C12 cells. Samples were collected and analyzed by PCR and agarose electrophoresis. The size of the target band amplified by PCR was approximately 400 bp, which is consistent with the theoretical value of 411 bp (Fig. 1A). The PCR amplification products were sequenced and a comparative analysis of the DNA sequencing results revealed that sgRNA3 had a greater effect on ADAM8 gene compared with the wild-type C2C12 cells (Fig. 1B). Western blot analysis further revealed that the expression of ADAM8 in mutant C2C12 cells was significantly decreased (*P* < 0.01). Therefore, ADAM8-sgRNA3 was considered as the most effective sgRNA (Fig. 1C). The agarose gels and sequencing data of plasmids in the supplements revealed that the ADAM8-sgRNA3 plasmid was successfully constructed.

### Successful construction of a mouse model of ALF

**Fig. 2:**
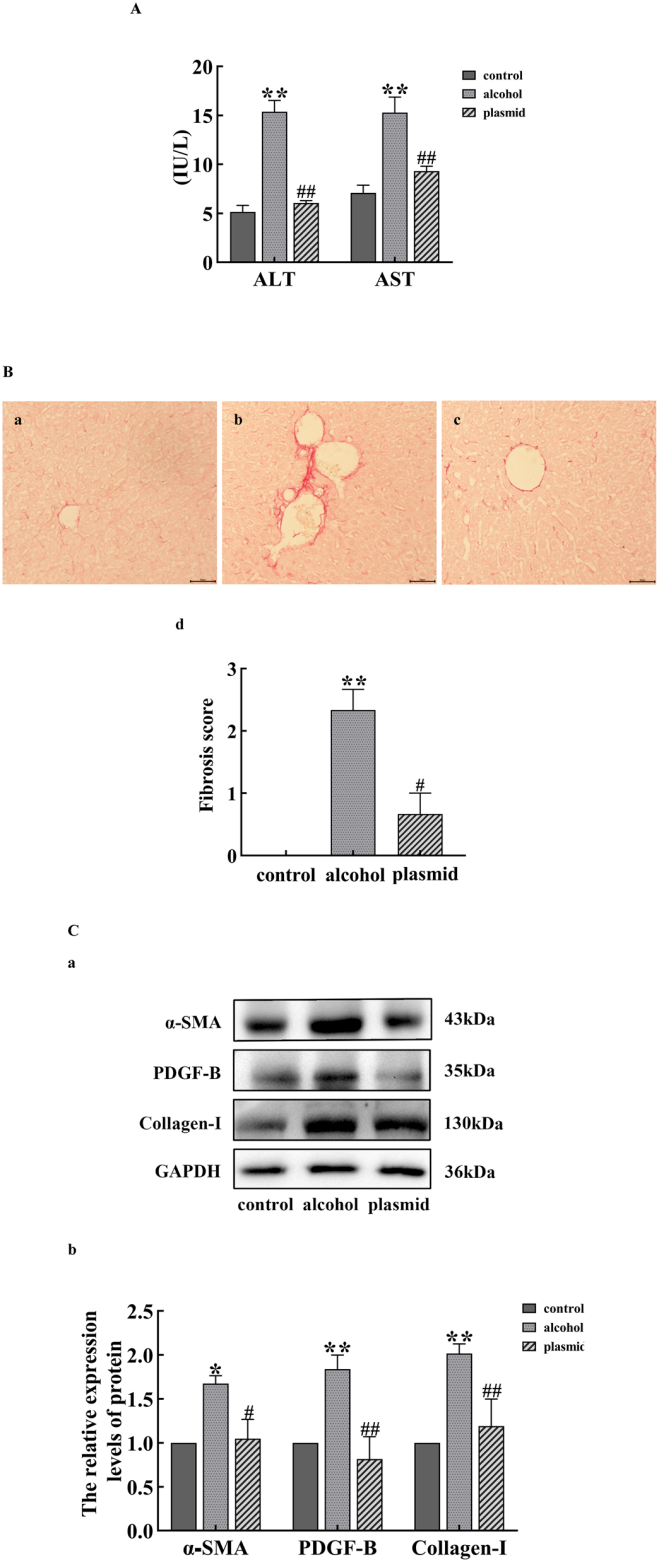
Validation of a mouse model of alcoholic liver fibrosis. **A** Changes in ALT and AST activity in each group of mice. **B** Detection of collagen fibers in the liver tissue of mice in each group. The Sirius Red staining charts for the control group (**a**), alcohol group (**b**), and ADAM8-sgRNA3 plasmid group (plasmid group) (**c**); The quantitative statistical chart of collagen fibers in the liver tissue of mice from each group (**d**). **C** Expression of α⁃SMA, PDGF-B, and Collagen-I proteins in the liver tissues of mice from each group. The immunoblot bands of α⁃SMA, PDGF-B, and Collagen-I proteins in the liver tissues of mice from each group (**a**). Quantitative statistical chart of α⁃SMA, PDGF-B, and Collagen-I protein expression changes in the liver tissues of mice from each group (**b**) (compared with the control group, ^*^P < 0.05 or ^**^P < 0.01; compared with the alcohol group, ^#^*P* < 0.05 or ^##^*P* < 0.01)

Compared with the control group, the enzyme activities of ALT and AST, the fibrosis score, and the expression of α-SMA, PDGF-B, and Collagen-I proteins in the alcohol group were significantly increased (*P* < 0.05 or *P* < 0.01). Compared with the alcohol group, the enzyme activities of ALT and AST, the fibrosis score, and the expression of α-SMA, PDGF-B, and Collagen-I proteins in the ADAM8-sgRNA3 plasmid group were significantly decreased (*P* < 0.05 or *P* < 0.01), as shown in Fig. 2.

### The expression of ADAM8 mRNA, protein and positive area rate in liver tissue of mice

**Fig. 3:**
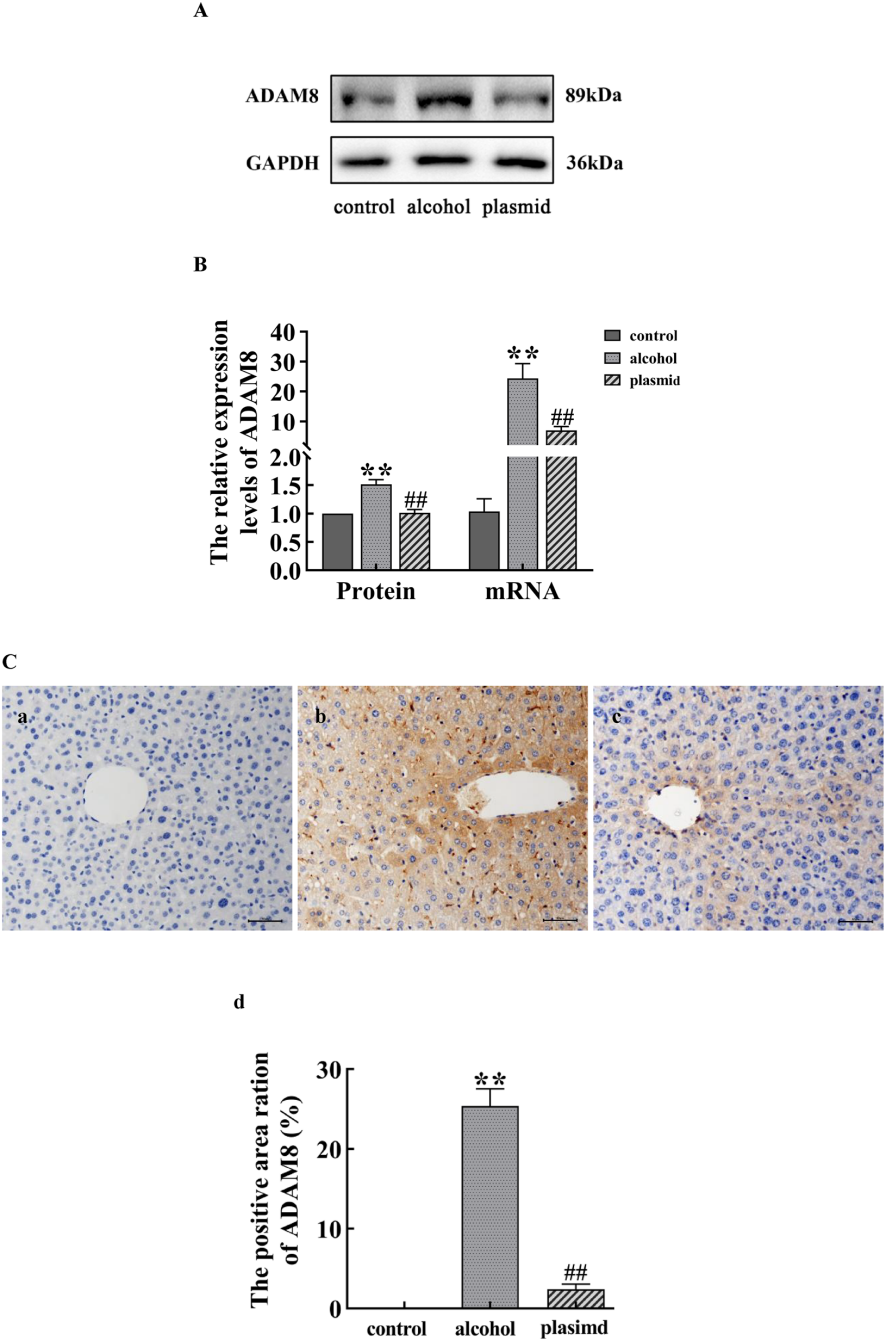
Expression of ADAM8 protein and mRNA in the liver tissue of mice from each group. **A** Immunoblot bands of ADAM8 protein in liver tissues of mice from each group. **B** Quantitative statistical chart of ADAM8 protein and mRNA expression changes in liver tissues of mice from each group. **C** Detection of ADAM8 expression of liver tissue in each group of mice using Immunohistochemistry. Staining charts for the control group (**a**), alcohol group (**b**), and ADAM8-sgRNA3 plasmid group (plasmid group) (**c**); Quantitative statistical charts for the liver tissue of mice in each group (**d**) (compared with the control group, ^**^P < 0.01; compared with the alcohol group, ^##^*P* < 0.01)

Compared with the control group, the expression of ADAM8 mRNA, protein and positive area rate in the liver tissue of mice in the alcohol group was significantly increased (*P* < 0.01), whereas the expression of ADAM8 mRNA, protein and positive area rate in the ADAM8-sgRNA3 plasmid group was decreased compared with the alcohol group (*P* < 0.01), as shown in Fig. 3.

### The effect of inhibiting ADAM8 expression in ALF mice

**Fig. 4:**
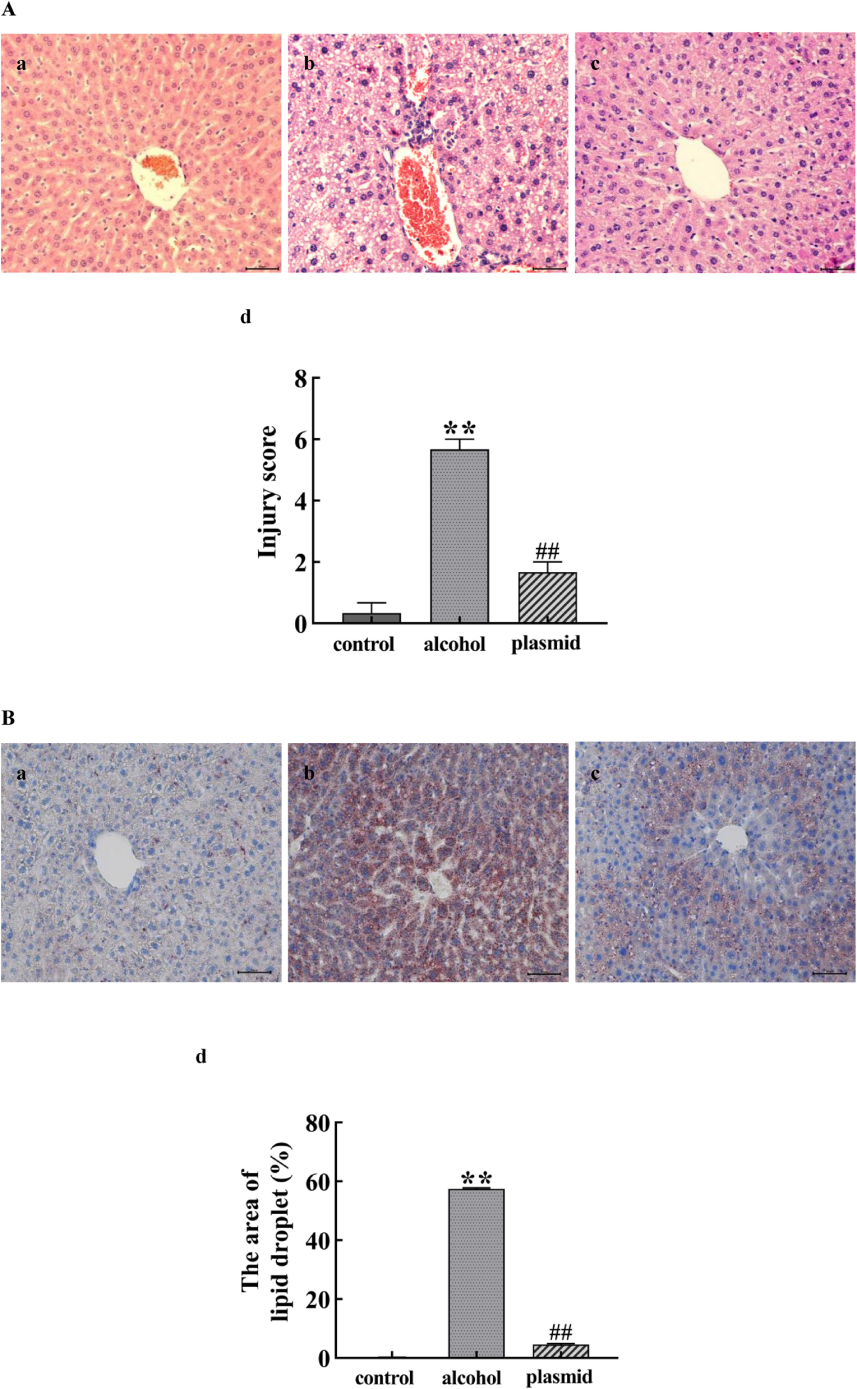

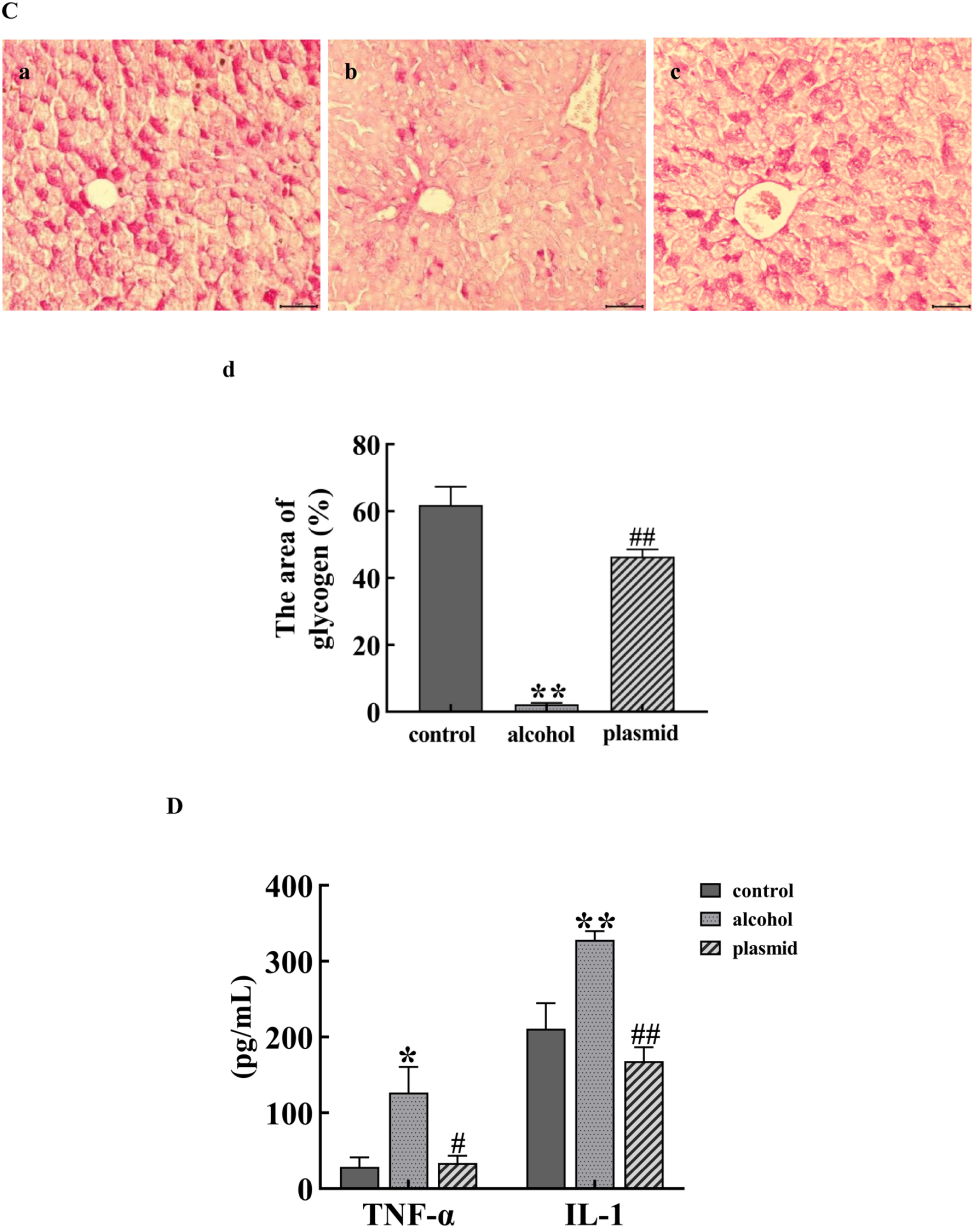
Effect of inhibiting ADAM8 expression in alcoholic liver fibrosis mice. Detection of liver tissue damage (**A**), lipid droplet content (**B**), glycogen content (**C**), and TNF-α and IL-1 (**D**) in each group of mice. Staining charts for the control group (**a**), alcohol group (**b**), and ADAM8-sgRNA3 plasmid group (plasmid group) (**c**); Quantitative statistical charts for the liver tissue of mice in each group (**d**) (compared with the control group, ^*^P < 0.05 or ^**^P < 0.01; compared with the alcohol group, ^#^*P* < 0.05 or ^##^*P* < 0.01)

Compared with the control group, the liver injury score, lipid droplet area rate, and the concentration of TNF-α and IL-1 in the alcohol group mice were significantly increased (*P* < 0.05 or *P* < 0.01), whereas the area rate of glycogen was significantly decreased (*P* < 0.01). The liver injury score, lipid droplet area rate, and the concentration of TNF-α and IL-1 in the ADAM8-sgRNA3 plasmid group were significantly reduced compared with the alcohol group (*P* < 0.05 or *P* < 0.01), whereas the area rate of glycogen was significantly increased (*P* < 0.01), as shown in Fig. 4.

### The mechanism of inhibiting ADAM8 expression in mice with ALF

**Fig. 5:**
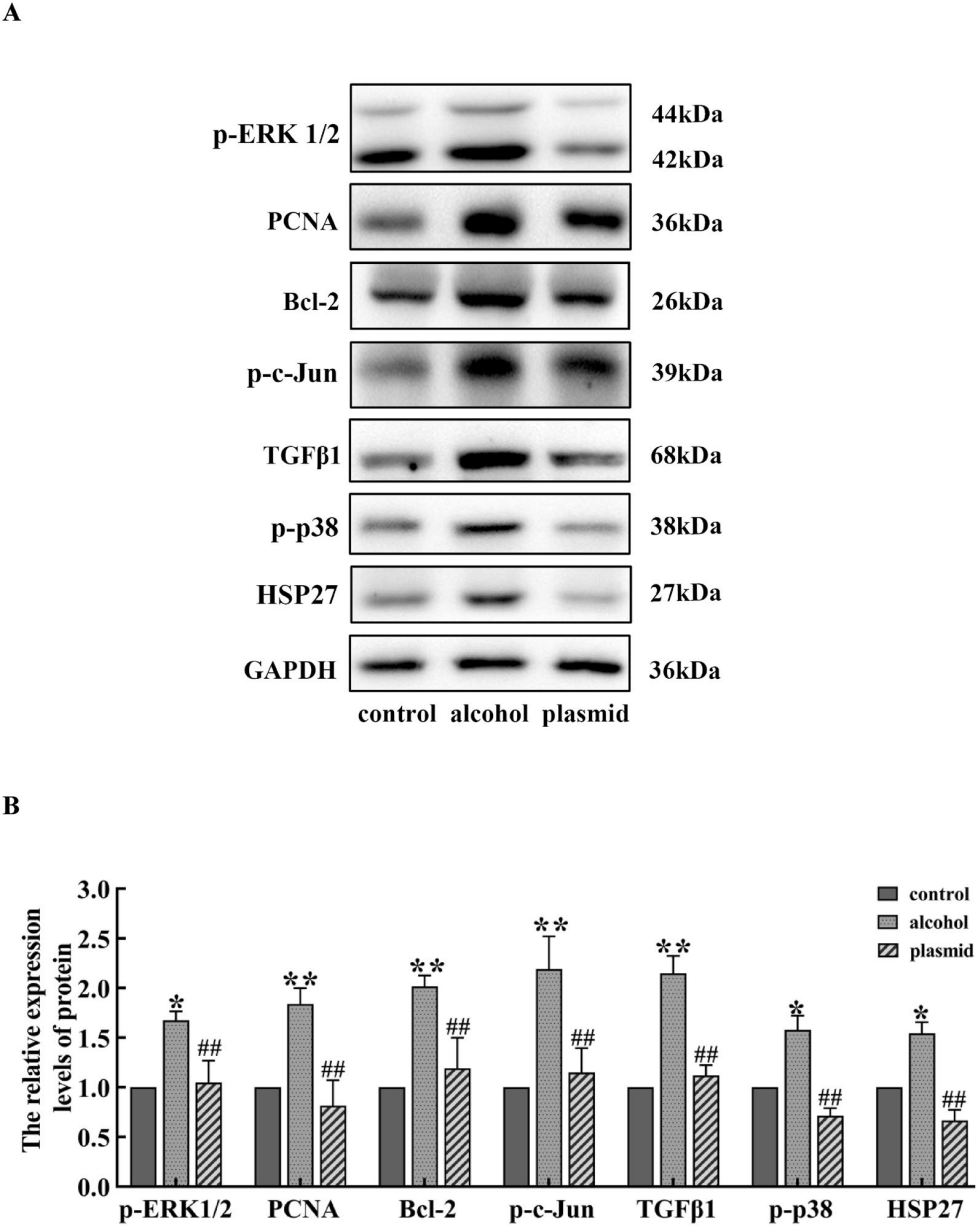
Expression of p-ERK1/2, PCNA, Bcl-2, p–c-Jun, TGFβ1, p–p38 MAPK and HSP27 proteins in liver tissue of mice from each group. **A** Immunoblot bands of p-ERK1/2, PCNA, Bcl-2, p–c-Jun, TGFβ1, p–p38 MAPK, and HSP27 proteins in liver tissues of mice from each group. **B** Quantitative statistical chart of the expression changes of p-ERK1/2, PCNA, Bcl-2, p–c-Jun, TGFβ1, p–p38 MAPK, and HSP27 proteins in the liver tissues of mice from each group (compared with the control group, ^**^P < 0.01 or ^*^P < 0.05; compared with the alcohol group, ^##^*P* < 0.01 or ^#^*P* < 0.05)

Compared with the control group, the expression of p-ERK1/2, PCNA, Bcl-2, p–c-Jun, TGFβ1, p–p38 MAPK, and HSP27 protein in the liver tissue of mice in the alcohol group was significantly increased (*P* < 0.05 or *P* < 0.01). Compared with the alcohol group, the expression of p-ERK1/2, PCNA, Bcl-2, p–c-Jun, TGFβ1, p–p38 MAPK, and HSP27 proteins in the ADAM8-sgRNA3 plasmid group was significantly decreased (*P* < 0.05 or *P* < 0.01), as shown in Fig. 5.

### Successful construction of LX-2 cell model for ALF

**Fig. 6:**
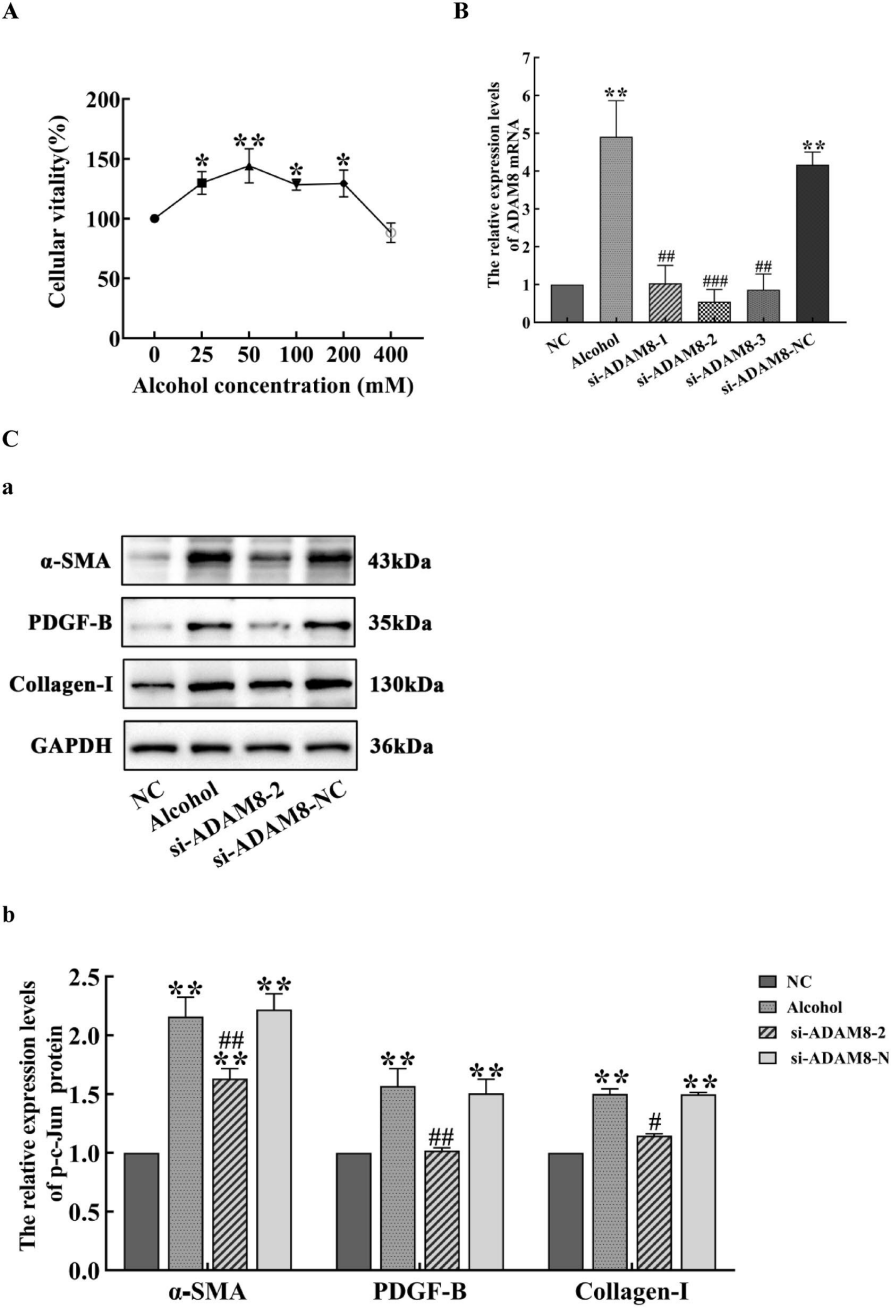
Validation of an LX-2 cell model for alcoholic liver fibrosis. **A** Effect of various concentrations of ethanol on the proliferation of LX-2 cells. Ethanol was added to the culture medium at concentrations of 0, 25, 50, 100, 200, and 400 mM (compared with untreated LX-2 cells, ^*^P < 0.05 or ^**^P < 0.01). **B** Effect of different siRNAs targeting ADAM8 (si-ADAM8s) on ADAM8 mRNA expression in LX-2 cells for each group. NC is the control group; Alcohol is the alcohol group; si-ADAM8-1, si-ADAM8-2, si-ADAM8-3, and si-ADAM8-NC were transfected into si-ADAM8-1, si-ADAM8-2, si-ADAM8-3, and si-ADAM8-NC groups, respectively (compared with the NC group, **P < 0.01; compared with the Alcohol group, ^##^*P* < 0.01 or ^###^*P* < 0.001). **C** Expression of α⁃SMA, PDGF-B, and Collagen-I proteins in LX-2 cells for each group. The immunoblot bands of α⁃SMA, PDGF-B, and Collagen-I proteins in LX-2 cells for each group (**a**). Quantitative statistical chart showing the expression changes in α⁃SMA, PDGF-B, and Collagen-I proteins in LX-2 cells for each group (**b**) (compared with NC group, ^*^P < 0.05 or ^**^P < 0.01; compared with the alcohol group, ^##^*P* < 0.01 or ^#^*P* < 0.05)

To stimulate LX-2 cells and assess the effect of ethanol on the viability of activated HSC in vitro, the LX-2 cells were exposed to varying concentrations of ethanol for 48 h. Cell viability of LX-2 cells at ethanol concentrations of 25, 50, 100, and 200 mM was significantly increased compared with the controls (*P* < 0.05). The vitality of LX-2 cells at an ethanol concentration of 50 mM was significantly increased (*P* < 0.01), as shown in Fig. 6A. Thus, the cells were exposed to an ethanol concentration of 50 mM for 48 h to establish an in vitro ALF model.

Three specific siRNAs (si-ADAM8-1, si-ADAM8-2, and si-ADAM8-3) were selected to inhibit the expression of ADAM8 in LX-2 cells. Quantitative RT-PCR was used to measure the relative expression of ADAM8 mRNA. The results indicated that the expression of ADAM8 mRNA in the alcohol and si-ADAM8-NC groups was significantly increased compared with that of the control group  (*P* < 0.01). Compared with the alcohol group, the expression of ADAM8 mRNA in the si-ADAM8-1, si-ADAM8-2, and si-ADAM8-3 groups was significantly decreased (*P* < 0.01). Of these, si-ADAM8-2 had the greatest effect at silencing ADAM8 gene expression (*P* < 0.001), as shown in Fig. 6B.

Compared with the control group, the expression of α⁃SMA, PDGF-B, and Collagen-I proteins in the alcohol group and si-ADAM8-NC group was significantly increased (*P* < 0.01), whereas they were significantly decreased in the si-ADAM8-2 group compared with the alcohol group (*P* < 0.05 or *P* < 0.01), as shown in Fig. 6C.

### The expression of ADAM8 protein and cell proliferation in LX-2 cells of each group

**Fig. 7:**
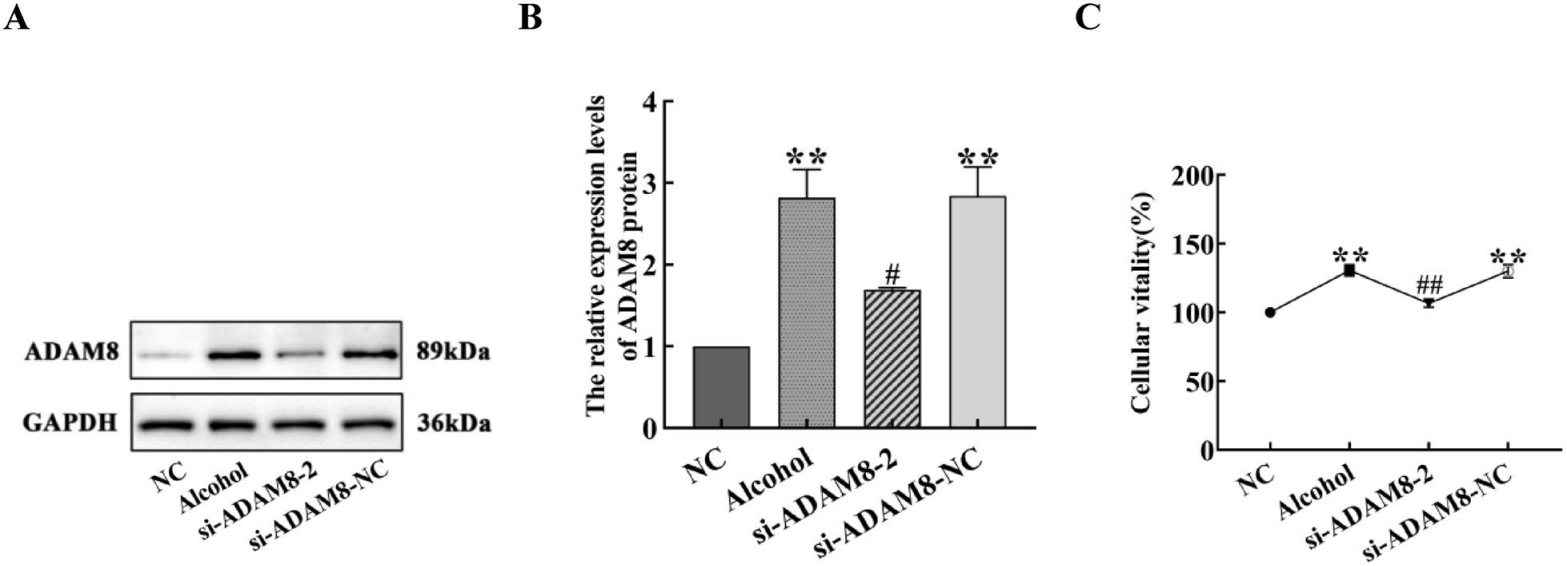
Expression of ADAM8 protein and proliferation of LX-2 cells in each group. **A** Immunoblot bands of ADAM8 protein in LX-2 cells for each group. **B** Quantitative statistical chart of ADAM8 protein expression changes in LX-2 cells for each group. **C** Proliferation of LX-2 cells in each group (compared with the NC group, **P < 0.01; compared with the alcohol group, ^##^*P* < 0.01 or ^#^*P* < 0.05)

Compared with the control group, the expression of ADAM8 protein and the proliferation of LX-2 cells in the alcohol and si-ADAM8-NC groups were significantly increased (*P* < 0.01). The expression of ADAM8 protein and the proliferation activity in the si-ADAM8-2 group was significantly decreased compared with the alcohol group (*P* < 0.05), as shown in Fig. 7.

### The mechanism of inhibiting ADAM8 expression in an alcoholic liver fibrosis model of LX-2 cells

**Fig. 8:**
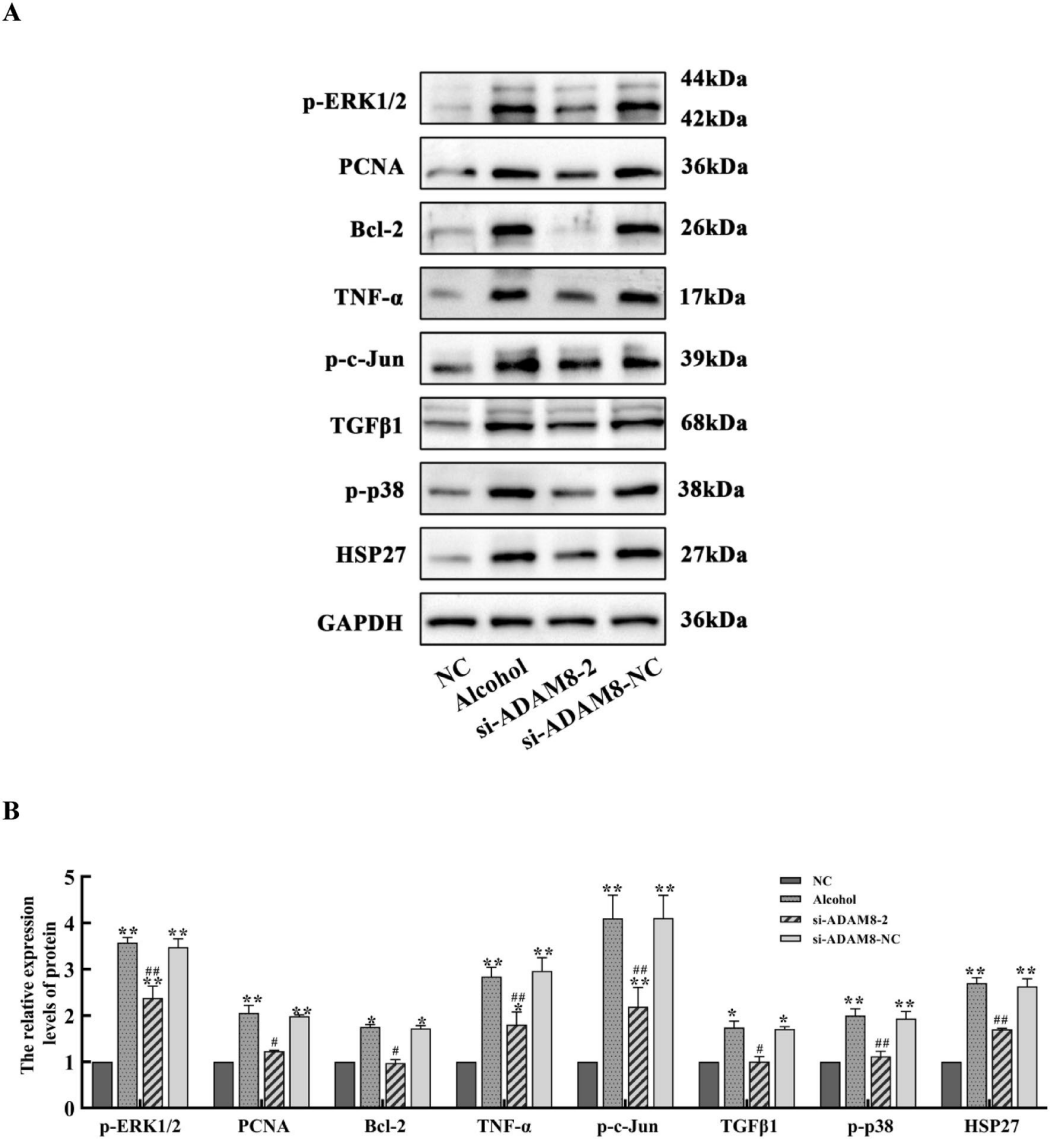
Expression of p-ERK1/2, PCNA, Bcl-2, TNF-α, p–c-Jun, TGFβ1, p–p38 MAPK, and HSP27 proteins in each group of LX-2 cells. **A** Immunoblot bands of p-ERK1/2, PCNA, Bcl-2, TNF-α, p–c-Jun, TGFβ1, p–p38 MAPK, and HSP27 proteins in each group of LX-2 cells. **B** Quantitative statistical chart of expression changes in p-ERK1/2, PCNA, Bcl-2, TNF-α, p–c-Jun, TGFβ1, p–p38 MAPK, and HSP27 proteins for each group of LX-2 cells (compared with the NC group, ^**^P < 0.01 or ^*^P < 0.05; compared with the alcohol group, ^##^*P* < 0.01 or ^#^*P* < 0.05)

Compared with the control group, the expression of p-ERK1/2, PCNA, Bcl-2, p–c-Jun, TGFβ1, p–p38 MAPK, TNF-α, and HSP27 proteins in the alcohol and si-ADAM8-NC groups was significantly increased (*P* < 0.05 or *P* < 0.01). Compared with the alcohol group, the expression of these proteins in the si-ADAM8-2 group was significantly decreased (*P* < 0.05 or *P* < 0.01), as shown in Fig. 8.

## Discussion

When alcohol continues to act on the liver, prolonged inflammatory stimulation activates liver macrophages, which secrete various pro-inflammatory and fibrotic factors, such as TNF-α, IL-1, and TGF-β1. This causes the continuous damage, repair, and compensatory proliferation of liver cells, as well as a large amount of α-SMA and Collagen-I secreted by activatived HSCs. The results in Fig. 2 indicate that the mouse model of ALF was successfully established by providing the animals with alcoholic liquid feed combined with 31.5% ethanol orally. The results in Fig. 6C indicate that the LX-2 cell ALF model was successfully established by treatment of cells with 50 mM ethanol for 48 h. The results in Figs. 4 and 7C further confirm the successful construction of an ALF model in vivo and in vitro.

In Figs. 3, 6B, and 7A and B, the expression of the ADAM8 protein and mRNA in the alcohol group was higher in vitro and in vivo, along with the positive area rate of ADAM8 in vivo, which suggests that ADAM8 may play an important role in promoting ALF. Moreover, Next, we determined the effect of inhibiting the expression of ADAM8 on ALF. The ADAM8-sgRNA3 plasmid was constructed to inhibit ADAM8 expression by CRISPR/Cas9 technology and injected into the tail vein of mice. The si-ADAM8-2 was transfected into activated LX-2 cells in vitro. After demonstrating the effective inhibition of ADAM8 expression, the degree of ALF was significantly decreased in Figs. 2 and 6C, indicating that ADAM8 is an important pro-fibrotic factor in ALF.

Daniela et al. found that ADAM8 has a pro-inflammatory function in acute lung inflammation using ADAM8-deficient mice. This suggests that high expression of ADAM8 is associated with the inflammatory reaction [18]. The expression of ADAM8 induced by various stimuli can hydrolyze and release the extracellular functional regions of membrane surface proteins, whereas secreted cytokines can affect the specific binding of ligands, thereby affecting signal transduction. There are also literature reports that chronic alcohol exposure can render Kupffer cells sensitive to endotoxin activation, thereby enhancing the production of the pro-inflammatory factors, TNF-α and IL-1β, which leads to inflammation, ECM production, and ALF. The results in Figs. 2D, 5, and 8 are similar to the above reports, suggesting that ADAM8 promotes an inflammatory response and exacerbates ALF by enhancing the expression of inflammatory mediators. However, the expression of inflammation-related cytokines decreased after inhibiting the expression of ADAM8, which indicates that inhibiting ADAM8 expression can relieve ALF by ameliorating the inflammatory response. In addition, the results of Figs. 4A–C and 7C further demonstrate that ADAM8 may be an effective target for treating ALF.

The MAPK signaling pathway is primarily involved in oxidative stress and the inflammatory response, whereas alcohol-induced oxidative stress and the inflammatory response are key steps in the ALF process. Oxidative stress activates the ERK signaling pathway, thereby promoting the proliferation and activation of HSCs [21, 22]. The ERK signaling pathway is also activated by PDGF, which promotes collagen production and deposition and induces liver fibrosis [23–25]. The increased levels of inflammatory mediators can activate the MAPK signaling pathway to promote the progression of liver fibrosis. TGFβ1 is considered the most important regulatory factor in promoting the formation of liver fibrosis [26–30] In addition to regulating the classical Smad pathway, TGFβ1 also activates p38 MAPK signaling pathway to mediate liver fibrosis and HSC activation [31, 32]. TNF-α promotes TGFβ1-induced HSC activation to affect the progression of liver fibrosis. IL-1β can also promote the activation and proliferation of HSCs through the p38 MAPK and JNK signaling pathways as well as the synthesis of Collagen-I [31, 32] to enhance the progression of liver fibrosis [33, 34].

ADAM8 silencing can inhibit IL-1-induced migration and invasion of fibroblast-like synovial cells through the MAPK cascade [35]. ADAM8 can enhance the invasiveness of glioblastoma by activating MAPK signaling [36]. Studies of ADAM8 and liver disease have also increased recently. The expression of ADAM8 is positively correlated with the activation of MAPK and regulates the proliferation and migration of liver cancer cells [37]. However, there is no definitive study showing a correlation between the expression of ADAM8 and MAPK signaling pathway during the occurrence and development of ALF. It will be important to determine if ADAM8 is involved in regulating the MAPK signaling pathway during the ALF process. In the present study, the expression of ADAM8 and MAPK signaling pathway-related factors was higher during alcohol-induced liver fibrosis. The results indicate that ADAM8 may promote ALF by activating the MAPK signaling pathway. After effectively inhibiting ADAM8 expression, changes in the MAPK signaling pathway were reversed. These results further demonstrate that downregulating ADAM8 expression ameliorates ALF by inhibiting the MAPK signaling pathway.

## Conclusions

**Fig. 9:**
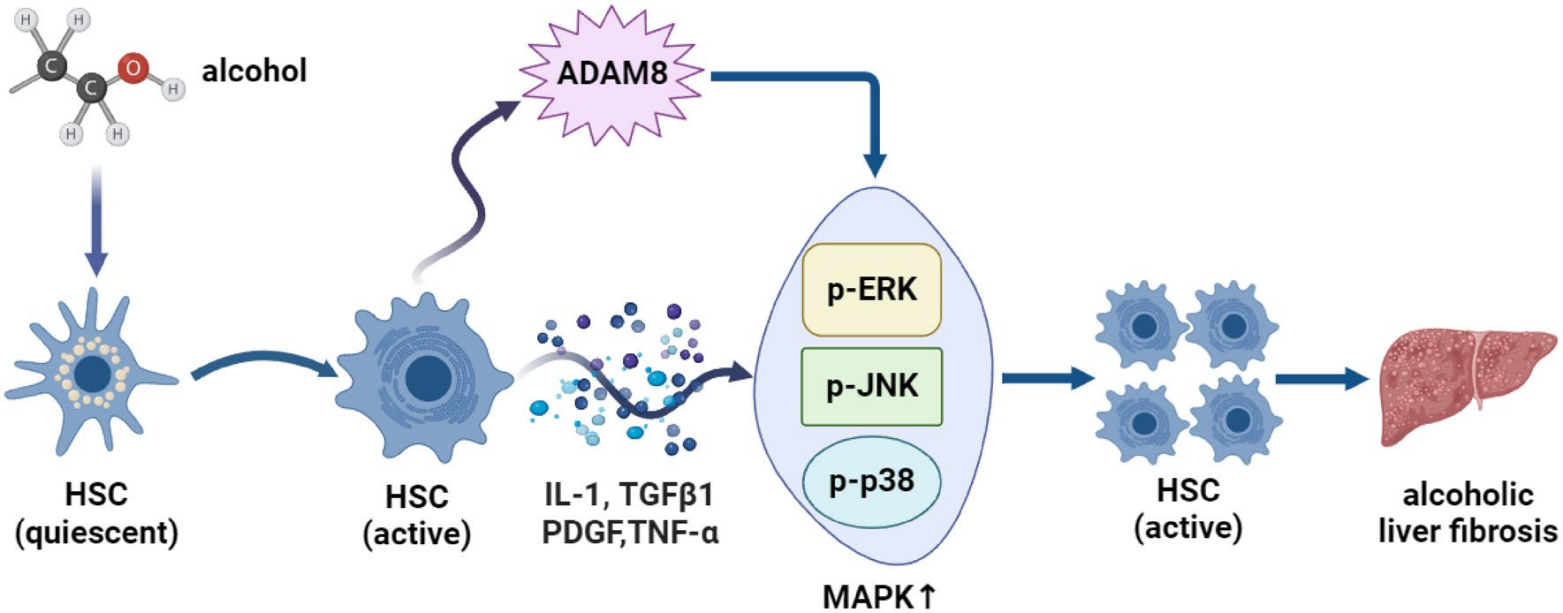
Mechanism diagram about how ADAM8 contribute to alcoholic liver fibrosis (ALF). Chronic alcohol consumption leads to the activation of hepatic stellate cells (HSCs), transitioning them from a quiescent state to an active state. In their active state, these HSCs begin producing pro-inflammatory and pro-fibrogenic cytokines, including IL-1, TGFβ1, PDGF, and TNF-α. ADAM8 expression is upregulated in response to these inflammatory signals, which subsequently activates the MAPK signaling pathway, including the phosphorylation of ERK, JNK, and p38 MAPK. This signaling cascade promotes further activation and proliferation of HSCs, which accelerates the fibrogenic process and contributes to the development of alcoholic liver fibrosis

In this study, ADAM8 had an important promoting effect on ALF in mice. Downregulating the expression of ADAM8 by CRISPR/Cas9 technology in vivo and siRNA in vitro can improve ALF by inhibiting the MAPK signaling pathway. The identification of this molecular mechanism (Fig. 9) indicates that ADAM8 may represent an effective biological target for the treatment of ALF.

## Supplementary Information

Supplementary material 1.

Supplementary material 2.

## Data Availability

All the data and materials are available.

## Abbreviations

ADAM: A disintegrin and a metalloproteinase
ALF: Alcoholic liver fibrosis
MAPK: Mitogen-activated protein kinase
ERK: Extracellular signal-regulated kinase
p-ERK: Phosphorylated extracellular signal-regulated kinase
JNK: C-Jun N-terminal kinase
p–c-Jun: Phosphorylated c-Jun
p–p38 MAPK: Phosphorylated p38 MAPK
ECM: Extracellularmatrix
MMP: Matrix metalloproteinase
TIMP: Tissue inhibitor of metalloproteinase
HSC: Hepaticstellate cells
α-SMA: α-Smooth muscle actin
PDGF-B: Plateletderived growth factor B
PCNA: Proliferating cell nuclear antigen
Bcl-2: B-cell lymphoma 2
TGF-β: Transforming growth factor β
HSP27: Heat shock 27
TNF: Tumor necrosis factor
IL-1: Interleukin-1
