# Supplementary material for: ADAM8 promotes alcoholic liver fibrosis through the MAPK signaling pathway
Source: J Physiol Sci. 2024 Oct 16;74:52. doi: 10.1186/s12576-024-00943-2 (PMC11481351; doi:10.1186/s12576-024-00943-2)
Supplement: Supplementary file 2 — Supplementary material 2. [file 12576_2024_943_MOESM2_ESM.docx]

**D**


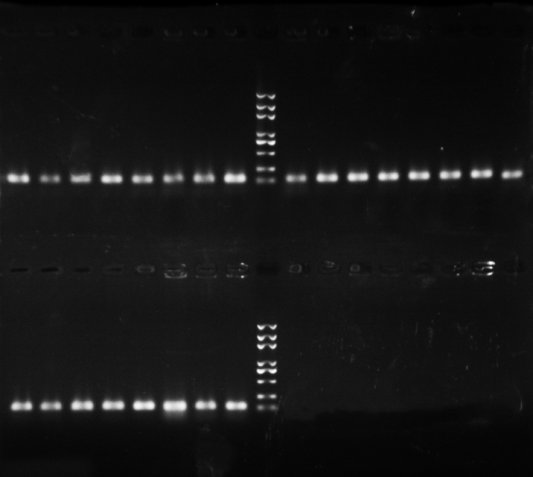


**a b**

**c**

**E**

a


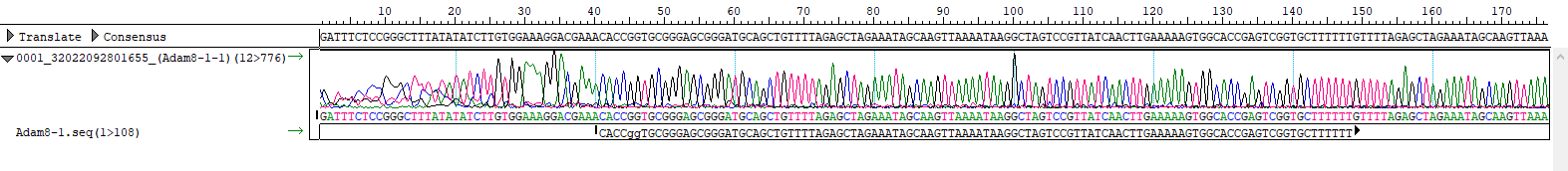


b


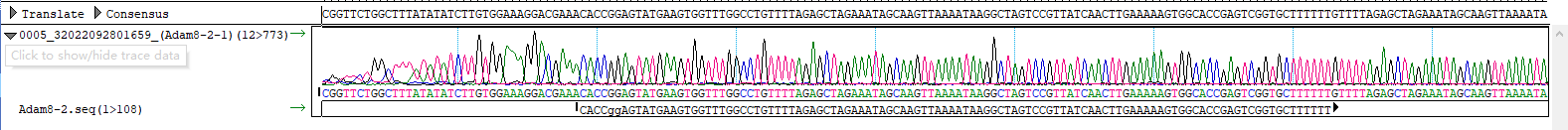


c


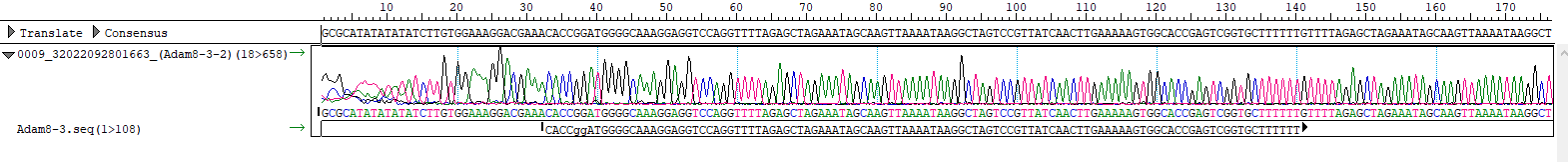


D. Agarose gel electrophoresis of positive clones validated by PCR of the transformants. The 9th lane from left to right is the DNA Marker, the molecular weights from top to bottom are 5000 bp, 3000 bp, 2000 bp, 1000 bp, 750 bp, 500 bp, 250 bp, and 100 bp, respectively. The plasmid DNA clones for ADAM8-sgRNA1 (a), ADAM8-sgRNA2 (b), and ADAM8-sgRNA3 (c). E. Transformation sequencing comparison results for pYSY-CMV-Cas9-U6-ADAM8-sgRNA1-EFla-puro (a), pYSY-CMV-Cas9-U6-ADAM8-sgRNA2-EFla-puro (b) and pYSY-CMV-Cas9-U6-ADAM8-sgRNA3-EFla-puro (c).

**Abbreviations**

| Abbreviations | Full name |
| --- | --- |
| ADAM  ALF  MAPK  ERK  p-ERK  JNK  p-c-Jun  p-p38 MAPK | A Disintegrin and A Metalloproteinase  Alcoholic liver fibrosis  Mitogen-activated protein kinase  Extracellular signal-regulated kinase  phosphorylated Extracellular signal-regulated kinase  c-Jun N-terminal kinase  phosphorylated c-Jun  phosphorylated p38 MAPK |
| ECM | Extracellularmatrix |
| MMP  TIMP  HSC | Matrix metalloproteinase  Tissue inhibitor of metalloproteinase  Hepaticstellate cells |
| α-SMA  PDGF-B  PCNA | α-Smooth muscle actin  Plateletderived growth factor B  Proliferating Cell Nuclear Antigen |
| Bcl-2 | B-cell lymphoma 2 |
| TGF-β | Transforming growth factor β |
| HSP27 | heat shock 27 |
| TNF  IL-1 | tumor necrosis factor  interleukin-1 |
